# MRI-guided laser interstitial thermal therapy in epilepsy: indications, technique and outcome in an adult population. A single-center data analysis

**DOI:** 10.1007/s00701-025-06429-3

**Published:** 2025-02-08

**Authors:** Nazaret Infante, Gerardo Conesa, Carmen Pérez-Enríquez, Jaume Capellades, Luísa Panadés de Oliveira, Laura Vilella, Alessandro Principe, Maria del Mar Crespi-Vallespir, Mireia Gallardo-Mir, Rodrigo Rocamora

**Affiliations:** 1 Department of Neurosurgery, Hospital del Mar, Barcelona, 08003 Spain; 2 Epilepsy Monitoring Unit, Department of Neurology, Hospital del Mar, Barcelona, Spain; 3 Epilepsy Neuroscience Research Group, Hospital del Mar Medical Research Institute (IMIM), Barcelona, Spain; 4 Neuroradiology Unit, Hospital del Mar, Barcelona, Spain; 5 StereoDive Medical SL, Barcelona, Spain; 6 Universitat Pompeu Fabra, Barcelona, Spain

**Keywords:** Drug-resistant epilepsy, Minimally invasive surgery, Laser ablation, MRgLITT, Cognition

## Abstract

**Background:**

Magnetic Resonance Imaging guided Laser Interstitial Thermal Therapy (MRIgLITT) is a promising treatment for drug-resistant epilepsy (DRE) and an alternative to open surgery. However, the relationship between clinical and radiological factors and postoperative outcomes is unclear. This study explores the indications, technical challenges, and outcomes of MRIgLITT in terms of seizure control and cognitive changes across various pathologies.

**Methods:**

A retrospective single-center analysis included 32 MRIgLITT procedures performed between January 2019 and December 2023. Procedures used the Visualase^®^ system for laser ablation, with stereotactic robotic guidance for fiber placement. Data included demographics, clinical and surgical details (ablated volume, timing, power and accuracy), and postoperative follow-up assessed seizure outcomes and complications. Cognitive changes were analyzed using a Reliable Change Index (RCI) before and one year after the procedure.

**Results:**

The 32 procedures involved 28 patients with MRI-diagnosed pathologies: 14 hippocampal sclerosis (HS), 7 hypothalamic hamartoma (HH), 3 focal cortical dysplasia (FCD), 2 periventricular heterotopia (PVH), 1 tuberous sclerosis complex (TSC), and 1 low-grade glioma. Some cases required multiple approaches.

Postoperative follow-up averaged 33 months. Among HS patients, 71.42% achieved Engel I, and 21.43% Engel II. In HH, 85.7% initially became gelastic seizure-free, with complete freedom after additional treatment. Engel I outcomes were 28.6%, while 57.2% showed significant improvement (Engel I + II). FCD patients had a 66.6% Engel I success rate. One PVH patient became seizure-free, while the TSC patient was Engel III at last follow-up. RCI analysis showed that 71.44% of patients experienced cognitive stability (RCI > −1.64) or improvement (RCI > 1.64) at one-year post-procedure.

**Conclusions:**

MRIgLITT is a safe, minimally invasive alternative for epilepsy surgery, offering quicker recovery and showing better performance preserving cognitive function. It is particularly effective for deep or complex epileptic foci and patients who might refuse open surgery.

**Supplementary Information:**

The online version contains supplementary material available at 10.1007/s00701-025-06429-3.

## Introduction

Epilepsy is a prevalent condition affecting approximately 3% of people over their lifetime, with around 30% experiencing drug-resistant epilepsy (DRE) [24]. Surgery represents the most promising option for achieving seizure freedom in many DRE patients [39, 46]. In recent decades, significant efforts have focused on developing minimally invasive techniques. Minimally invasive approaches primarily target focal epilepsies, which constitute about 60% of all epilepsy cases [8, 30]. These techniques require a well-defined epileptogenic focus. In ablative procedures, the objective is to destroy the epileptogenic lesion when present and disrupt the epileptogenic network, which requires a precise identification and delineation of the seizure onset zone (SOZ) through prior diagnostic studies. Furthermore, these procedures aim to maximize function preservation in surrounding healthy tissue, emphasizing the principle of highly selective surgical lesioning.

Magnetic Resonance Imaging guided Laser Interstitial Thermal Therapy (MRIgLITT) has been increasingly applied as a first-line surgical treatment for focal epilepsy in recent decades. The technique enables real-time MRI monitoring of the thermal lesion during the procedure [33]. It operates on the principle where high-intensity light converts into heat energy within a defined area, monitored via magnetic resonance thermometry. During the procedure, the temperature is continuously monitored at specified marked points on the thermal map. The software employs a model to estimate the ‘irreversible damaged area’ based on the relationship between time and temperature, overlaying this information on anatomical images. This approach ensures accurate control over tissue damage with precision in the order of millimeters, providing instant feedback [19, 22, 23, 33, 52].

Several key advantages include its capacity to treat larger volumes compared to other ablative techniques [43], avoidance of craniotomy, rapid patient recovery, and the ability to minimize or entirely prevent damage to cortical areas when reaching target [1, 5, 7, 10, 27–29, 31, 36, 41, 44, 48, 53], making it possible to treat deep epileptogenic lesions and near eloquent areas.

However, the relationship between clinico-radiological factors and postoperative outcomes is unclear. There is no data from large prospective multicenter studies, and it is necessary to unify the indications and standardize the surgical technique to determine which parameters and target volumes yield the best results with the fewest complications.

This article aims to present our center’s experience with MRIgLITT, including its indications, technical challenges, outcomes, and strategies for preventing potential complications. Additionally, we share insights gained during the initial years of using this technique, as one of the first centers to implement it in our country. Specifically, we focus on cognitive changes observed across a wide range of indications, addressing a gap in the currently available information.

## Methods and materials

At the Epilepsy Unit of Hospital del Mar, European Reference Network EpiCARE center, the presurgical evaluation protocol includes a 3T T1-weighted MRI (1 mm slices), video electroencephalography (vEEG), neuropsychological evaluation and when needed additional studies such as ictal/interictal SPECT, PET, VBM imaging-postprocessing, electrical source imaging, functional MRI or Wada test for language dominance.

The results of the presurgical studies are evaluated by a multidisciplinary committee. The MRIgLITT technique is indicated when the seizure semiology, the onset of electrical activity in the vEEG recording, MRI findings, cognitive profile, and functional studies, if performed, are concordant.

For patients with temporal lobe epilepsy (TLE) and hippocampal sclerosis (HS), MRIgLITT is primarily recommended for the dominant hemisphere due to high economic costs, reserving the technique to minimize cognitive impairment. In hypothalamic hamartoma (HH), it is the technique of choice in all cases. In other indications, its use is preferred when lesions are difficult to access for open resective surgery. In some cases, it is the only technique that patients accept to undergo surgery.

### Patient selection

This study includes all consecutive MRIgLITT procedures performed in our institution, between January 2019 and December 2023. Patients with less than 6 months of follow-up were excluded. Data, retrospectively collected, included patient demographics and clinical data such as age at surgery, age at epilepsy onset and time until surgery, seizure frequency, anti-seizure medication (ASM) and radiological diagnoses, surgical details included the number of fibers, ablation time, power used and accuracy, complication records and outcomes (at 3 months, 6 months, 1 year, 3 years and last follow-up). Seizure postoperative outcomes were assessed according to Engel Scale criteria [14] and complications were collected from clinic notes. For patients undergoing a second surgery, the last follow-up outcomes for the ablation were obtained prior to subsequent surgeries. All our patients were evaluated with a pre-surgical cognitive protocol and re-assessed at 3 and 12 months after surgery. Details of the neuropsychological evaluation protocol were previously published [34]. To determine the significant cognitive changes, the algorithm of the Reliable change index (RCI) [13] was implemented.

### Surgical technique and procedure details

Specific protocols were created for each the pathology responsible for epilepsy (HS, HH, FCD, PVH and tumours), including catheter type, target location, safety points location, and specific issues, such as premedication in HH. The goal was to standardize the procedure, particularly at the start of the MRIgLITT program.

All cases begin with an initial test to confirm the maximal heat point, which helps define the “continuous temperature monitoring” points.

Temporal lobe epilepsy: In HS, we use a 10 mm diffusing tip, with safety points adjusted during the procedure depending on the ablation site. These points are fixed to protect the third cranial nerve and mesencephalon during hippocampal head ablation, as well as the thalamus and optic radiations posteriorly during hippocampal tail ablation. The fiber is retracted by 7 mm in each ablation to achieve uniform area coverage.

Hypothalamic hamartoma: In HH, a 3 mm diffusing tip fiber is typically used; however, a 10 mm tip may be necessary for large lesions, such as in Delalande type IV. The presurgical evaluation includes campimetry and a hormonal profile study. All HH patients are premedicated with 4 mg dexamethasone every 6 h during the week prior to surgery. The trajectory was designed avoiding the ventricles, the optic tract, fornix, mammillothalamic tract and mammillary bodies.

Other indications: In other indications, such as FCDs or PVHs, the direction of the catheter or the combined use of more than one fiber was defined according to the specific anatomy of the lesion. In any case, 10 mm catheters were always used.

**Fig. 1:**
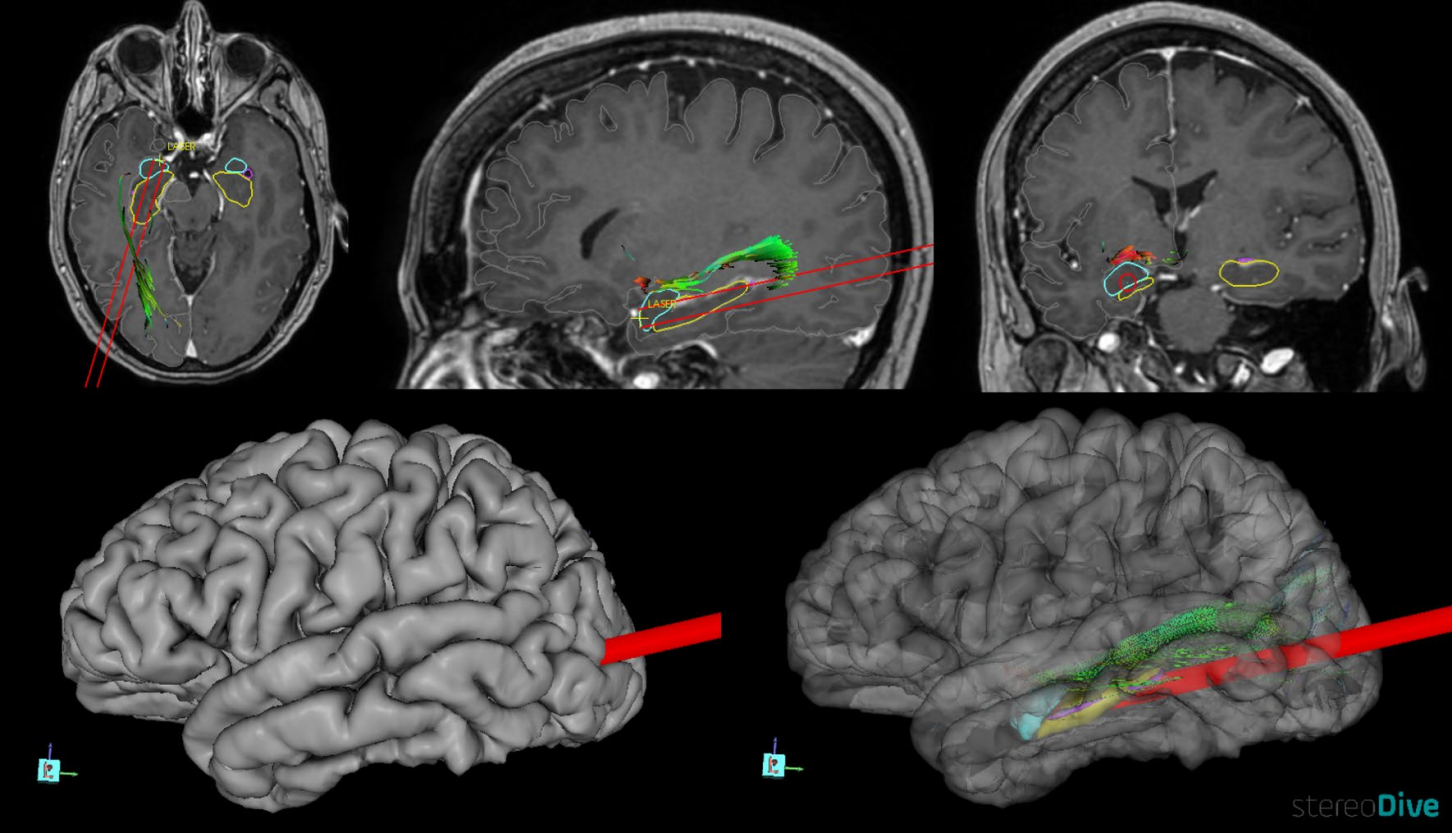
3D planning imaging in a HS case. Trajectory planning is performed using a Fig. 1. 3T MRI with double contrast and SD tractography, using StereoDiver® software

Design of trajectories: Trajectory planning is performed using a 3T MRI with double contrast and spherical deconvolution (SD) based tractography from diffusion-weighted imaging (DWI) sequence, using StereoDiver^®^ software [21] and finally validated with Neuroinspire, Renishaw^®^ software (Fig. 1). Vascular structures were considered in all cases.

Catheter implantation: The procedure is conducted under general anesthesia. The Leksell stereotactic coordinate frame (Elekta AB, Stokholm, Sweden) is used for skull fixation, and a 0.6 mm slice head CT scan is done for coregistration with the previously planned MRI sequence. Fiber placement was guided by the Rosa^®^ robot in nine initial procedures, and by Renishaw^®^ robot in subsequent ones; titanium anchor bolts are used except for superficial targets like focal cortical dysplasia (FCD), where plastic bolts are used to avoid artifacts.

**Fig. 2:**
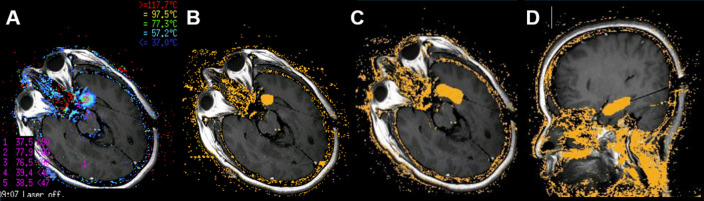
Monitoring images during the procedure in a HS case. Continuous temperature monitoring at specified marked points on the thermal map using magnetic resonance thermometry (**A**). The software employs a model to estimate the ‘irreversible damage area’ based on the relationship between time and temperature, overlaying this information on two selected planes (**B** , **C** and **D**), marked in orange

**Fig. 3:**
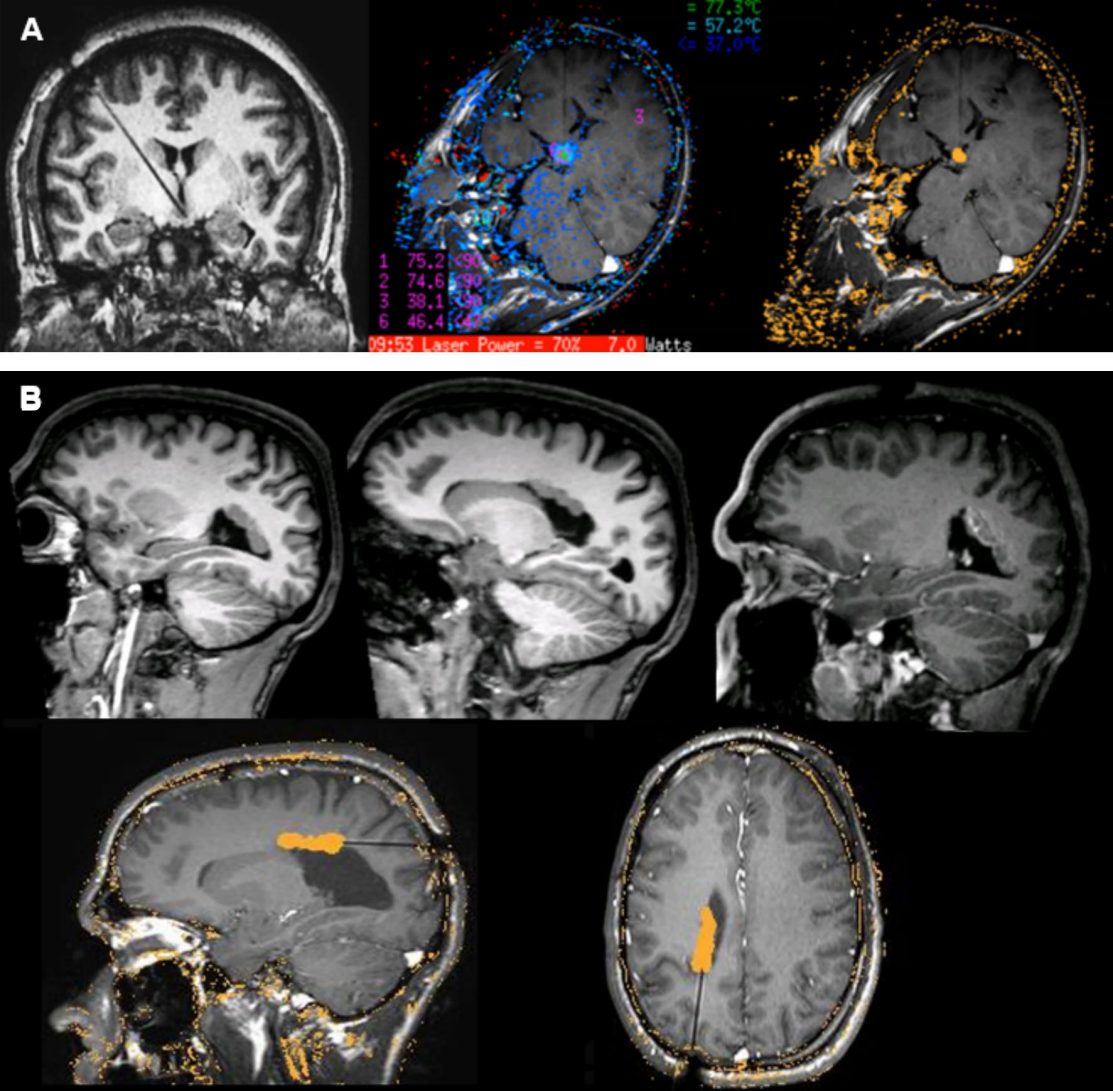
Monitoring images during the ablation procedure. T1 sequence confirming correct fiber placement, temperature monitoring on thermal map and estimated ablation volume in a HH case (**A**). C-shaped PVH case treated with a two-time approach. Presurgical sagittal T1 MRI, final contrast-MRI sequence post-ablation after first approach, and estimated ablation volume during the second procedure (**B**)

Ablation procedure: After removing the Leksell frame, the patient is transferred to the MRI room for an initial MRI to confirm correct laser probe placement before starting the ablation. The Visualase^®^ system from Medtronic is employed, delivering laser energy through a 15 W, 980 nm diode laser, using a saline-cooled catheter to deliver a fiber optic with a 3 or a 10 mm diffusing tip, depending on the target lesion. Real-time thermal imaging via MRI monitors the procedure, ensuring safety by setting limits that automatically shut off the laser if exceeded. Laser energy is adjusted during the procedure to maintain target temperatures between 60–85ºC, based on real-time temperature observations and the estimated “irreversible damage area” (Figs. 2 and 3).

The ablation is controlled through a station, with continuous temperature monitoring at selected points. Two markers are placed near the fiber to prevent temperature from exceeding 90ºC, which could damage it, and three markers at nearby tissue and structures at risk with a low limit of 47ºC to prevent thermal effects. This allows marking the security zone with millimetric precision.

Four patients (12.5%) received treatment with more than one laser fiber in the same approach, a second-time procedure was also required in three patients, two HH and a large C-shaped periventricular heterotopia (PVH).

A higher error with significant deviation from the planned trajectory was noticed in two consecutive cases coinciding with the robot system change (see on tables ID patients: #3 on his second approach and #11), without any associated complications. These cases were considered as outliers and not included in mean error measurements. After this, longer tool-holder adapters were acquired for the drilling step, and narrower ones for guiding the bolt placement, with a 1.7 mm diameter light, improving the accuracy of the procedure.

### Neuropsychological assessment protocol

All our patients are routinely evaluated with a pre-surgical cognitive protocol and reassessed at 3- and 12-months post-surgery. The details of the neuropsychological evaluation protocol were published previously [34]. The neuropsychological assessment includes a broad range of cognitive domains, such as attention, executive function, language, verbal and visual/figural memory, and visuospatial skills. The tests used for each domain, as well as the subtests analyzed in this study, are listed in Supplementary Table 4. For this study, only baseline (pre-surgical) data (T0) and follow-up data at one-year post-surgery (T2) were analyzed.

To determine significant individual cognitive changes, we applied the Reliable Change Index (RCI) algorithm [12] with a 90% confidence interval. The RCI is a widely used method for interpreting reliable changes in contexts like this, where repeated neuropsychological evaluations over time are required. This method helps to minimize or eliminate the learning effect that can occur when the same tests are administered repeatedly to the same individuals. RCI analysis is a subject-based approach, meaning it analyzes data on an individual, case-by-case basis, comparing each individual to a clinical control group (in our case, a database of patients with focal epilepsy who were evaluated pre-surgically and at a one-year follow-up while awaiting epilepsy surgery).

### Statistical analysis

A descriptive analysis was conducted for all the variables of interest. For numeric variables, the mean, standard deviation, and range were calculated, while categorical variables were summarized with absolute and relative frequencies.

The correlations between the seizure outcome, Engel Class at last-follow up, and each variable of interest (“Age at MRIgLITT”, “Epilepsy onset age”, “Seizure frequency”, “Dominance”, “Hemisphere”, “MRI diagnosis”, “HH subtype”, “Location”, “Ablation time”, “Power”, “% Planned-ablation volume”) were analyzed using chi-square or Fisher’s exact tests for categorical data, and Student’s t-tests for comparisons involving numeric data.

Statistical analysis was performed using the R statistical software (Version 4.4.1, R Foundation for Statistical Computing, 2024, https://www.R-project.org/) [37], with statistical significance set at 0.05.

## Results

### Population demographics

This study includes a total of 32 MRIgLITT procedures in 28 patients (16 male). Demographic data was collected and is shown in Table 1.

**Table 1:** Patients and clinical data. BRV Brivaracetam, CBZ Carbamazepine, CLB Clobazam, D daily, DZP diazepam, FCD focal cortical dysplasia, HH hypothalamic hamartoma (specifying delalande classification subtype), HS hippocampal sclerosis, LCM Lacosamide, LEV Levetiracetam, LTG Lamotrigine, M monthly, MRI dx diagnose by magnetic resonance imaging, N number, OXC Oxcarbazepine, PB Phenobarbital, PER perampanel, PGB Pregabalin, PreS ASM presurgical anti-seizure medication, PVH Periventricular Heterotopia, SCB Eslicarbazepine, SD several daily, SM several monthly, SV several weekly, TSC Tuberous sclerosis complex, TPM topiramate, TPO temporo-parieto-occipital, Tx Tumour, VPA valproic acid, W weekly, Y years, ZNS zonisamide

ID	Gender	Age at MRIgLiTT (y)	Age at epilepsy onset (y)	Dominance	Seizure frequency	Seizures/month (*n*)	PreS ASM (*n*)	ASM	Hemisphere	Location	MRI Dx
1	Male	52	16	Left	SM	5	3	ZNS, BRV, LTG	Left	Temporal	FCD
2	Female	36	16	Right	SW	12	5	LCM, CLB, LEV, PER, SCB	Left	Temporal	Tx
3	Male	28	9	Left	SW	14	3	LEV, PGB, CBZ	Right	Extratemporal	HH IIIB
4	Male	42	5	Right	SD	90	3	BRV, CBZ, PER	Left	Extratemporal	HH IIA
5	Male	25	9	Right	SW	7	3	SCB, LEV	Right	Extratemporal (TPO)	PVH
6	Female	33	13	Right	M	2	3	SCB, CLB, BRV	Left	Temporal	HS
7	Male	54	28	Right	SW	16	3	CBZ, PGB, PER	Left	Temporal	HS
8	Male	28	8	Right	SD	30	5	BRV, ZNG, LCM, PER, DZP	Left	Extratemporal (Frontal)	TSC
9	Male	52	1	Right	M	1	2	VPA, PB	Left	Temporal	HS
10	Female	30	17	Right	SM	4	5	BRV, CLZ, ZNS, CLB, SCB	Left	Extratemporal	HH IIIB
3	Male	30	3	Left	SM	5	3	LEV, PGB, CBZ	Right	Extratemporal	HH IIIB
11	Male	44	18	Right	W	20	3	LCM, BRV, CLB	Left	Temporal	HS
12	Male	30	2	Right	D	224	2	LEV, LTG	Right	Extratemporal (Frontal)	FCD
13	Female	24	9	Right	W	3	3	BRV, SCB, CLB	Right	Temporal	HS
14	Female	30	20	Right	SD	30	2	LEV, SCB	Left	Temporal	PVH
15	Female	33	0	Right	SD	140	3	SCB, ZNS, CLB	Right	Extratemporal	HH IV
16	Female	58	0	Right	D	30	3	LTG, CLB, ZNS	Left	Temporal	HS
17	Male	56	8	Right	M	3	2	CBZ, LCM	Left	Temporal	HS
5	Male	28	9	Right	W	8	3	CNB, LEV, CLB	Right	Extratemporal (TPO)	PVH
18	Male	36	24	Left	D	14	3	OXC, LTG, PER	Left	Temporal	HS
19	Female	25	1	Right	SW	14	5	LEV, LCM, ZNS, SCB, CLB	Left	Temporal	HS
20	Male	32	4	Right	SM	2	2	SCB, BRV	Left	Temporal	HS
21	Male	49	12	Right	SW	60	3	BRV, SCB, CNB	Left	Temporal	HS
3	Male	33	3	Left	SM	4	3	LEV, PGB, CBZ	Right	Extratemporal	HH IIIB
22	Female	27	6	Right	SM	3	3	LTG, TPM, LEV	Left	Temporal	HS
23	Male	30	20	Right	SM	3	2	CBZ, LCM	Right	Temporal	HS
24	Female	35	1	Left	SW	30	3	LEV, LCM, OXC	Left	Temporal	HS
15	Female	34	0	Right	SD	140	3	SCB, ZNS, CLB	Right	Extratemporal	HH IV
25	Female	25	3	Right	D	30	4	LEV, LTG, CBZ, CLB	Right	Extratemporal	HH IIIA
26	Male	21	7	Right	SD	70	3	LCM, BRV, DZP	Right	Extratemporal	HH IIIB
27	Male	45	6	Right	D	90	3	SCB, BRV, CLB	Left	Extratemporal (Frontal)	FCD
28	Female	62	7	Right	SW	30	2	BRV, CLB	Left	Extratemporal	HH IIIB

All treated cases were diagnosed with lesional DRE. Based on MRI diagnosis, there were 14 HS cases, mainly for dominant hemisphere (85.7% of cases in our sample), 7 HH, 3 FCD, 2 PVH, 1 of tuberous sclerosis complex (TSC), and 1 of glioma. Subsequent procedures were required four times: in two HH cases and in one PVH case. Age at surgery was 37.21 years (range 21 to 62 years), and age at epilepsy onset was 9.64 years. Therefore, there was a delay of 27.57 years from epilepsy diagnosis to surgery. Patients were taking a mean of 3.1 ASM at the time of surgery, which decreased to 2.8 at the last follow-up. Regarding seizure frequency, occurrences were daily in 35.71% of patients, weekly in 35.71%, and monthly in 28.57%. The mean follow-up after surgery was 33 months, with follow-up periods ranging from 8 to 67 months.

Overall, seizure control was favorable, but due to the diverse surgical indications, subgroup analysis is necessary.

### Surgical procedure measurements

To assess the accuracy and error of fiber insertion, each planned trajectory was compared to the final fiber placement. Differences were measured at both, the entry and target points, using Euclidean and radial distances, and analyzed through Stereodiver^®^ software (Table 2).

**Table 2:** Surgical procedure data. EE euclidean error, N number, NA not available, RE radial error

ID	Fibers (*n*)	Total ablation time (s)	Power (W)	Targeted volume (mm^3)	Ablated volume (mm3)	Planned ablated (mm3)	Intersection (%)	Entry EE (mm)	Entry RE (mm)	Target EE (mm)	Target RE (mm)	Complications
1	1	NA	NA	NA	NA	NA	NA	1,07	1,06	1,59	1,37	No
2	1	558	4,2	286,52	880,66	223,24	77,91	0,13	0,12	0,77	0,76	No
3	1	96	3,4	NA	NA	NA	NA	NA	NA	NA	NA	No
4	1	1355	4,1	277,67	231,39	189,78	68,35	2,02	2,01	3,57	2,54	No
5	1	1344	8,5	1.945,90	5.937,89	1.570,61	80,71	1,59	1,56	2,22	1,1	No
6	1	501	9,2	1.255,97	8.232,80	1.255,97	100	2,16	2,1	5,24	2,1	No
7	1	868	9,2	1.804,39	3.979,69	1.503,81	83,34	0,71	0,58	2,46	2,11	No
8	1	578	8,7	2.872,51	8.234,37	1.158,76	40,34	1,21	1,07	2,49	2,35	No
9	1	1556	9,2	3.036,45	9.751,50	2.434,47	80,17	2,53	2,53	1,56	1,46	Yes
10	1	130	NA	145,65	153,19	93,28	64,04	2,04	2,03	0,96	0,65	No
3	1	156	4	58,01	65,92	1,76	3,03	0,43	0,38	4,83	3,7	No
11	1	365	8,7	NA	NA	NA	NA	4,06	3,99	8,38	7,26	No
12	2	367	9,5	437,32	1.549,10	185,84	42,5	1,66	1,6	3,2	1,7	No
13	1	360	NA	3.252,52	4.250,85	1.067,65	32,83	1,92	1,88	4,24	3,98	No
14	1	111	7,5	503,35	428	372,99	74,1	0,85	0,73	1,06	0,83	No
15	1	256	6,5	2.566,14	1016,7	986,21	38,43	1,33	1,33	2,49	1,41	No
16	1	575	8,6	2.963,24	2.820,24	1.767,96	59,66	0,56	0,55	1,69	1,5	No
17	1	488	7,6	3.109,29	3.208,31	2.339,05	75,23	2,7	2,31	3,09	1,25	No
5	1	457	11,6	1.182,12	3.598,20	1.037,10	87,73	0,74	0,74	1,08	0,91	No
18	1	733	9,4	4.049,38	3.413,33	2.744,10	67,77	1,12	0,96	3,1	2,45	No
19	1	571	9,8	3.243,86	4.289,27	2.426,97	74,82	0,13	0,11	2,74	2,59	No
20	1	629	9,7	4.771,63	3.838,94	3.266,26	68,45	1,15	1,15	4,4	3,58	No
21	1	271	11,1	3.355,00	1.809,00	1.114,00	33,2	1,73	1,61	3,93	3,04	No
3	1	150	6,1	106	249	77	72,64	0,94	0,94	1,38	1,25	No
22	1	532	8,2	1.591,00	1.326,00	846	53,17	2,51	2,18	2,64	0,86	No
23	1	508	8	3061	3.436,00	1.980,00	64,68	1,08	0,76	2,56	1,6	No
24	1	697	8,5	4820,5	3.677,00	1934,5	40,13	1,39	1,27	1,78	1,65	No
15	2	1288	5,6	2774,98	1.437,99	1.115,99	40,22	0,46	1	2,52	1,78	No
25	1	157	5,9	360	818	359	99,72	1,31	1,25	1,79	0,84	Yes
26	1	61	4	152	205	105	69,08	1,29	1,27	1,39	1,33	No
27	2	910	7’2	2345,96	6.487,90	1.069,98	45,61	1,86	1,77	0,97	0,85	No
28	1	347	5,3	162	435	108	66,67	1,42	1,42	3,13	3,11	No

The mean error at the entry point was 1.33 mm (Euclidean distance) and 1.28 mm (radial distance), and at the target was 2.50 mm (Euclidean distance) and 1.84 mm (radial distance). The mean ablation time per case was 547.58 s, but it varied significantly when multiple ablations were required during the procedure, depending on the size of the treated lesion. Mean ablation volume was 3060.55 mm^3^. The mean time per each position was 176.14 s, and power used average was 7.58 W.

**Fig. 4:**
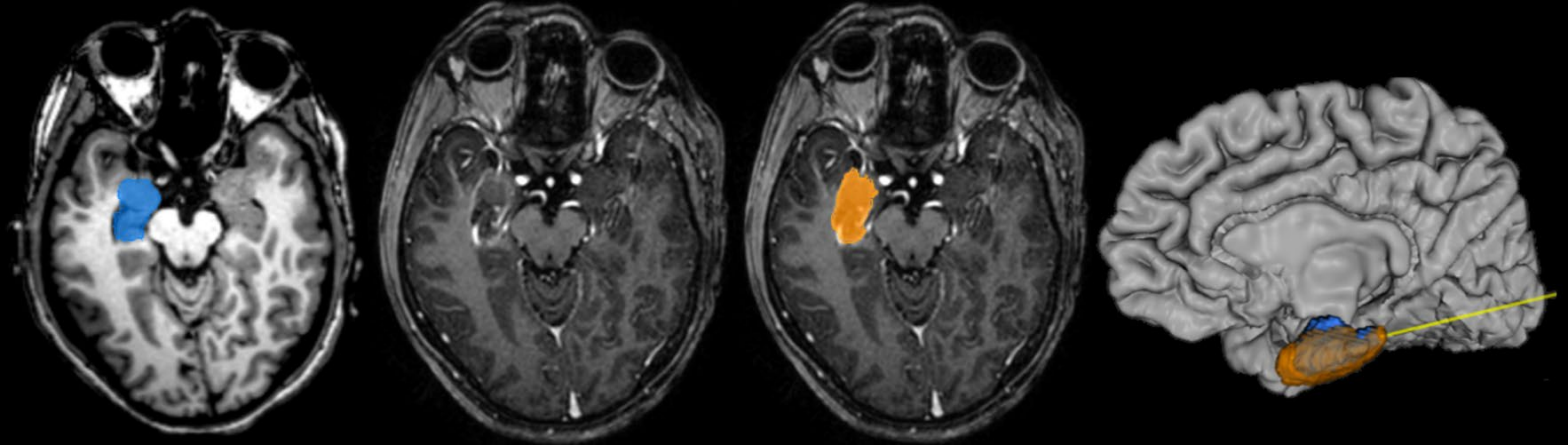
Postoperative MRI in a HS case. Final contrast-MRI sequence and segmentation of the planned (marked in blue) and ablated (marked in orange) volumes with StereoDiver ® for subsequent measurements, considering the internal ring of enhancement, showing the intersection in 3D volume

The ideal MRI-planned ablation volume on StereoDiver^®^ software was compared with the final ablated volume assessed on the postoperative contrast MRI (Fig. 4).

**Fig. 5:**
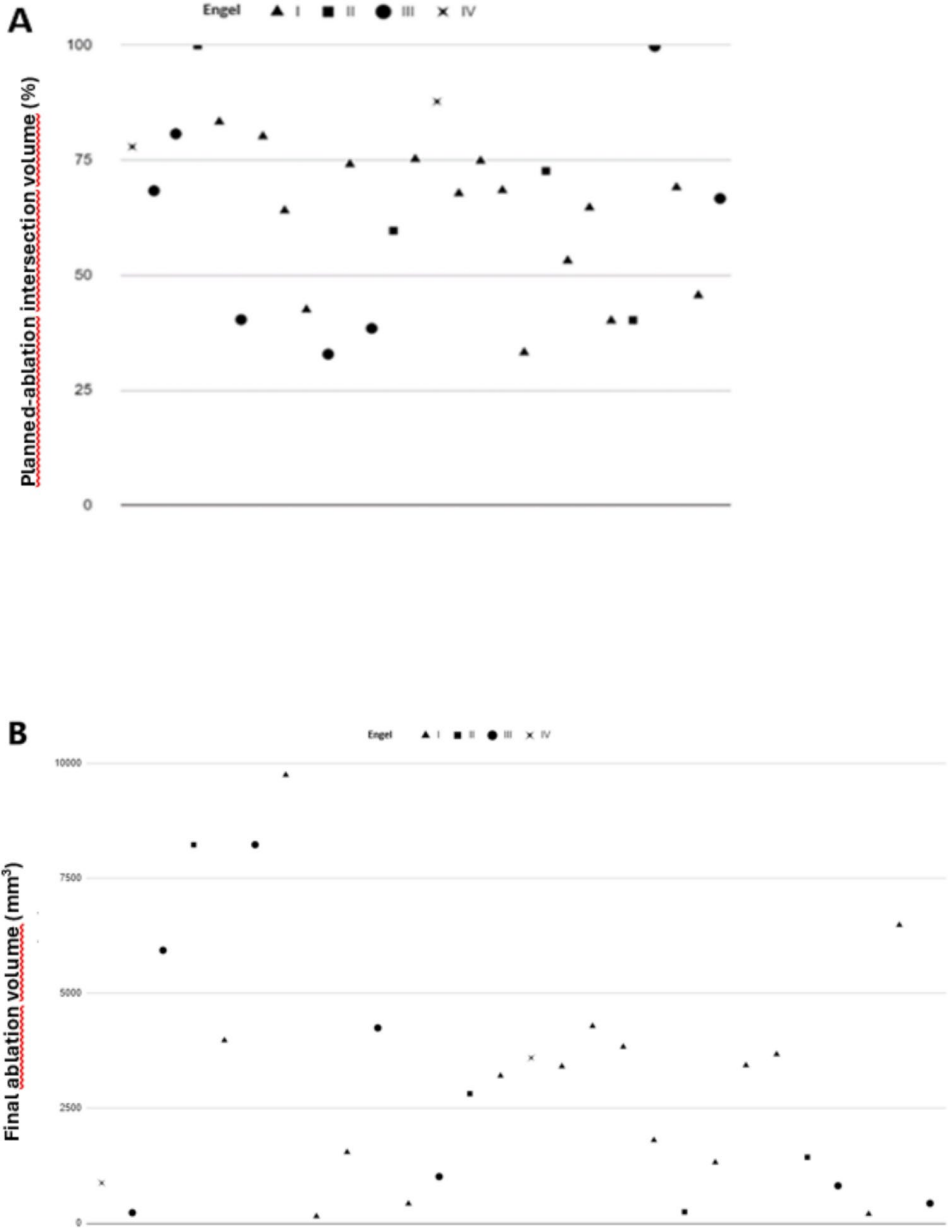
Scatterplot (**A**) Engel outcome at last follow-up related to planned-ablation intersection volume (%). Scatterplot (**B**) Engel outcome at last follow-up related to final ablation volume (mm3)

The final planned volume coverage was 64.34%. The accuracy of the final ablated volume identification remains unclear, the internal ring of enhancement was considered. Volumetric analysis was performed on all the ablations, and no difference was found between seizure-free and not seizure-free patients. There were no differences between Engel outcome and % planned-ablated volume or final ablated volume (Fig. 5).

#### Efficacy and seizure control outcome

Postsurgical outcomes, with a mean follow-up of 33 months, are shown on Table 3. In patients with HS, 71.42% achieved Engel Class I, 21.43% Engel Class II, and 7.14% Engel Class III. In FCD patients, 66.6% achieved Engel Class I, and 33.3% Engel Class II. For HH patients, 85.71% were free from gelastic seizures at first approach, turning into 100% of the cases after a second laser treatment. All types of seizures were controlled (Engel Class I) in 28.6% of HH patients, 28.6% were Engel Class II, and 42.9% Engel Class III. In PVH, one patient achieved seizure freedom, while the other did not improve significantly (Engel III). The TSC patient, previously confirmed by SEEG the predominantly involvement of a single tuber in his typical seizures, was classified as Engel III at the last follow-up. The patient with a tumor diagnosis had previously undergone open surgery for a temporal lobe astrocytoma resection. Due to persistent seizures, MRIgLITT for an amygdala remnant was proposed, and the patient was classified as Engel Class II at the last follow-up.

**Table 3:** Outcome and follow-up. ASM anti-seizure medication, M months, N number

ID	Follow up (m)	Engel at last follow up	Gelastic seizures	Seizure reduction (%)	Postsurgical ASMs (*n*)	ASM reduction
1	67	II	-	−100,00%	3	No
2	66	IV	-	−83,33%	3	Yes
3	65	III	Persistent	−92,86%	3	No
4	63	III		−66,67%	3	No
5	62	III	-	14,29%	3	No
6	59	II	-	−50,00%	2	Yes
7	58	I	-	−100,00%	2	Yes
8	57	III	-	−96,67%	6	No
9	47	I	-	−100,00%	1	Yes
10	44	I	Free	−100,00%	1	Yes
3	42	III	Free	−20,00%	3	No
11	39	II	-	−65,00%	2	Yes
12	38	I	-	−100,00%	1	Yes
13	33	III	-	−33,33%	5	No
14	28	I	-	−100,00%	3	No
15	27	III	Free	0,00%	3	No
16	26	II	-	−99,33%	3	No
17	23	I	-	−100,00%	2	No
5	22	IV	-	−12,50%	3	No
18	21	I	-	−100,00%	3	No
19	19	I	-	−100,00%	1	Yes
20	18	I	-	−75,00%	2	No
21	18	I	-	−99,83%	3	No
3	17	II	Free	−95,00%	3	No
22	16	I	-	−83,33%	3	No
23	15	I	-	−66,67%	2	No
24	15	I	-	0,00%	5	No
15	14	II	Free	−99,29%	4	No
25	11	III	Free	−93,33%	4	No
26	10	I	Free	−100,00%	3	No
27	9	I	-	−100,00%	3	No
28	8	III	Free	−96,67%	2	No

**Fig. 6:**
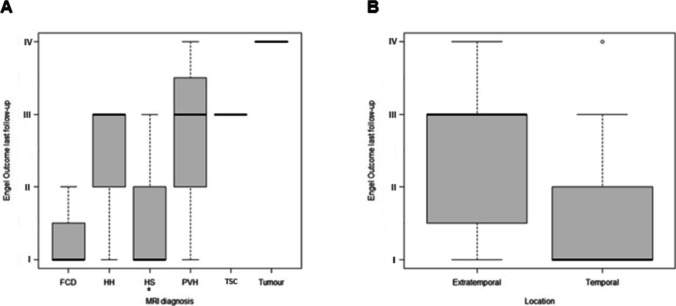
Boxplot (**A**) Engel outcome at last follow-up related to MRI diagnosis. Boxplot (**B**) Engel outcome at last follow-up related to location of lesion, temporal versus extratemporal

Outcomes were statistically significantly better in hippocampal sclerosis compared to the other pathologies. Good results were also observed in FCD, but due to the small sample size, it was not significant (Fig. 6A). Additionally, seizure control was significantly better in temporal than extratemporal epilepsies in our population (Fig. 6B). No associations between Engel class outcomes and the rest of the analyzed variables were found.

The total number of seizures per month on average, including all cases, decreased from 35.44 to 7.48, leading to a reduction of more than 75%. At the last follow-up, ASM could be reduced in 28.57% of MRIgLITT patients.

### Cognitive outcomes

All details of the baseline (T_0_) or 1-year post-Litt (T_2_) descriptive neuropsychological data can be reviewed in Supplementary Table 5. Analyses of individual reliable changes using the RCI revealed that up to 71.44% of the cases showed stability (no significant change, RCI>−1.64) or gain (RCI > 1.64) in the one-year post-MRIgLITT neuropsychological assessment.

**Fig. 7:**
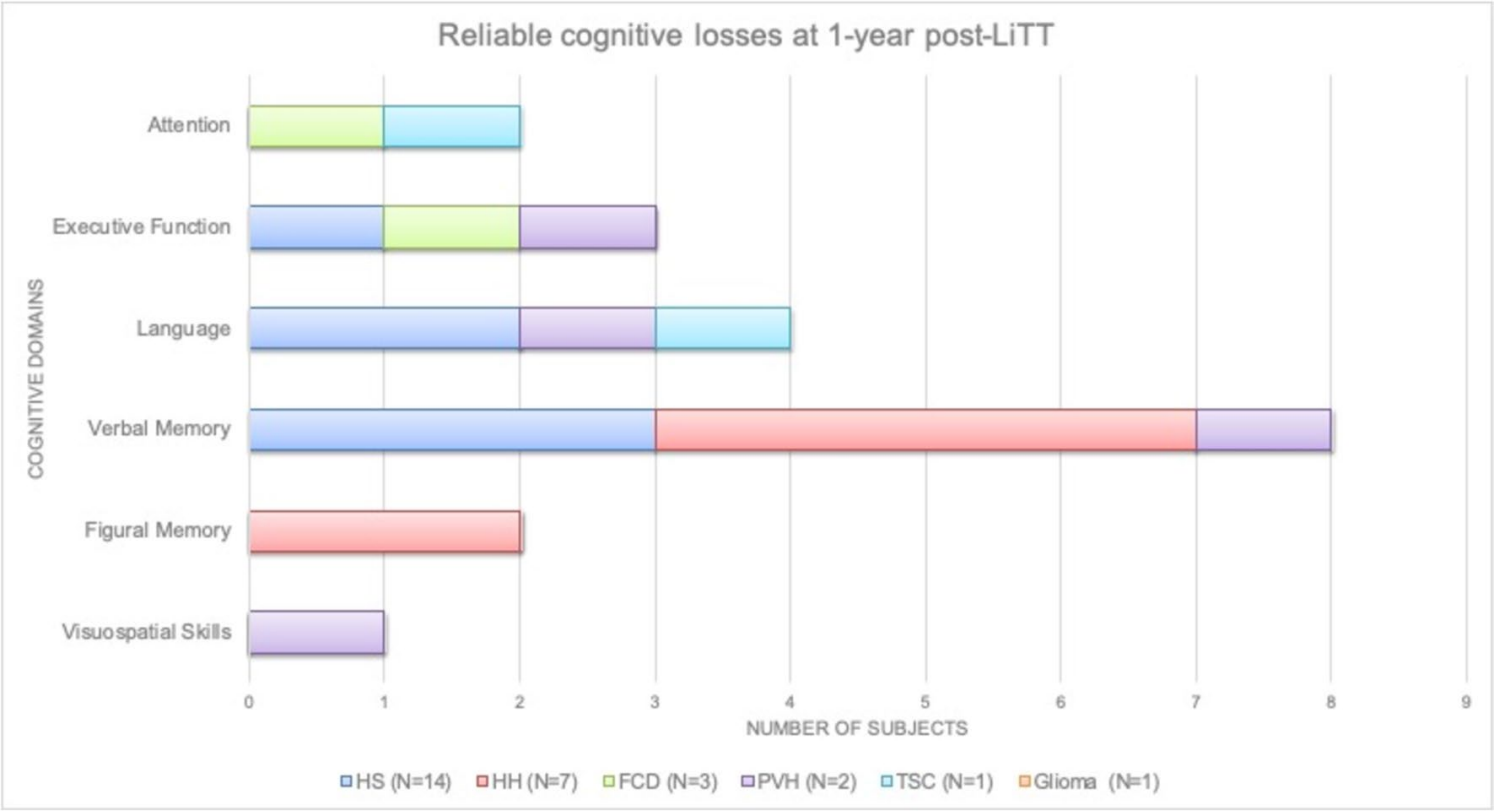
Reliable cognitive losses individual level-basis at 1-year post-LITT follow-up across different neuropsychological domains

Significant losses (RCI <−1.64) were found from highest to lowest frequency in: verbal memory (8 cases; according to MRI: *N* = 3 HS, *N* = 4 HH, *N* = 1 PVH), language (4 cases; *N* = 2 HS, *N* = 1 TSC, *N* = 1 PVH), visual/figural memory (3 cases; *N* = 3 HH), executive function (3 cases; according to MRI: *N* = 1 HS, *N* = 1 PVH, *N* = 1 FCD), attention (2 cases; *N* = 1 FCD; *N* = 1 TSC) and finally, visuospatial skills (a single case; *N* = 1 PVH). For the frequency distribution of individual reliable losses by type of MRI lesion, see Fig. 7.

According to MRI pathology, of the 14 patients who underwent surgery for mesial temporal lobe epilepsy (HS), 3 (21.42%) exhibited verbal memory deficits, 2 (14.28%) naming deficits and one case (7.14%) executive function. In addition, none of the patients with HS showed significant deficits in attention, visuospatial skills or visual memory. Of the subgroup of 7 patients who underwent surgery for hypothalamic hamartomas (HH), 4 (57.14%) exhibited cognitive deficit in verbal memory and two patients (28.57%) in figural memory. Among the 3 patients who underwent surgery for FCD, only one case (33.33%) showed deficits in attention and executive function. A single case of the 2 patients with PVH (50%), showed deficits in the following domains: executive function, language, verbal memory, and visuospatial skills. The only patient with TSC exclusively showed deficits in attention and language. No cognitive domains were affected after MRIgLiTT in the patient with glioma diagnosis.

### Postoperative complications

Two cases experienced complications related to the treatment (6.25% per procedure). In one patient treated with a mesial temporal ablation, hemianopsia was noted due to heat spreading posteriorly beyond the planned target, affecting the optic radiation. One case, among the HH group (10% per procedure) developed hyponatremia, behavioral changes, and memory deficits, as previously mentioned, that resolved over weeks. There were no instances of hemorrhage, infection, or cerebrospinal fluid leakage. All patients were discharged within 24–48 h after the procedure.

## Discussion

MRIgLITT is an increasingly used surgical technique in epilepsy treatment, primarily aimed at destroying the epileptogenic lesion, if present, and/or disrupting the epileptogenic network. Its successful implementation depends on the precise identification and delineation of the SOZ. This approach maximizes the preservation of function in surrounding healthy tissue while enabling real-time monitoring of thermal injury during the procedure [19, 22, 23, 33, 52].

As a minimally invasive surgical technique, MRIgLITT offers an alternative treatment option for high-risk surgical patients and could potentially increase referrals for surgery among patients with medically refractory epilepsy.

To date, there are few MRIgLITT series detailing laser ablation procedure including different etiologies, and these often involve small samples. The efficacy of MRIgLITT (58%) in temporal lobe epilepsy is comparable, although slightly lower than, the results of anterior temporal lobectomy (ATL) (64%). In comparison to the available literature, our results in HS group (71.42% in Engel Class I) suggest that seizure outcomes following MRIgLITT are similar to those reported after ATL, being around a 75% seizure-free at one-year follow-up after an ATL or amygdalohippocampectomy [1, 17–19, 25–27, 35, 44, 46, 50]. MRIgLITT is associated with fewer surgical complications compared to ATL, with a reported hemorrhage rate of 1.5% and a neurological complication rate of 11%, primarily involving visual field defects.

MRIgLITT also demonstrates a lower incidence of neuropsychological deficits in TLE, with reductions in verbal and visual memory of 24.2% and 25.2%, respectively, and a 13.4% decrease in naming ability. It is particularly recommended for patients at higher cognitive risk or those with higher surgical risk, such as older individuals. In the HS patients from our series, similar rates of verbal memory and naming deficits were observed, 7.14% exhibited executive function impairment, but none showed significant deficits in attention, visuospatial skills or visual memory.

Furthermore, the main advantages are its safety and quick neurological recovery, making it a viable alternative, particularly for patients with HS.

The two-fiber approach in HS has been described in the literature [32], the need for a double approach will definitely depend on the anatomy. Nevertheless, in our series of cases, coverage with a single probe has been sufficient in all instances. Additional comparative studies are needed to assess the impact of ablation volume on clinical outcomes, particularly given the high cost of each fiber.

MRIgLITT for HH has become a significantly safer alternative compared to other approaches, such as open or endoscopic surgery. MRIgLITT in HH showed a very positive result (21/25 Engel I = 84%) and low complication rate. HH with RF-CT shows an Engel I of 71% (71/100); in comparison, HH treated with radiosurgery showed an Engel I of 46.2% (24/52) without permanent neurological deficits; surgical series (52/107) only achieved an Engel I in 48% and a high complication rate (9–15%) [2, 20, 38, 47]. Staged ablative treatments may be necessary to achieve optimal results while minimizing damage, as disconnection often plays a more critical role than the ablation volume [16]. In our series, MRIgLITT demonstrated high efficacy in controlling gelastic seizures, with 100% of patients free from gelastic seizures -one requiring a second procedure- aligning with previously reported literature [49]. However, published studies often do not characterize the volume or morphology of the lesions, and most of these studies focus on pediatric population. In adults, it is common for patients to present with other seizure types at the time of surgery, and success rates considering these other seizure types, are not well described. Based on our findings, seizure freedom from all seizure types is less promising, we noted a 57.2% showing significant improvement (Engel I + II), but just 28.6% achieved Engel I, significantly lower compared to other series in pediatric population [47].

We hypothesize that the differences in seizure control rates between the current study and previous studies may be related to lesion morphology, particularly the attachment point, Delalande type, as well as secondary epileptogenesis [40]. Patients with larger lesions may require staged ablative treatment with multiple approaches to achieve complete disconnection. We observed a delay of 27 years from epilepsy diagnosis to surgery in our series, which could be a decisive factor. This supports the attitude toward early surgical intervention to achieve the best outcomes with this technique.

The average seizure freedom rate for FCD treated with MRIgLITT is 59% (95% CI: 44–74%), while the rate of achieving a ≥ 50% seizure reduction is 90% (95% CI: 80–100%). These outcomes are comparable to those observed with RF-TC. Meta-analyses indicate that MRIgLITT and RF-TC are associated with a lower incidence of postoperative complications compared to other techniques. However, MRIgLITT does not demonstrate superior efficacy or a lower complication rate when compared to traditional surgical resection [3, 42]. Our series, though limited in size, achieved seizure freedom (Engel Ia) in 2/3 treated patients. Cognitive outcomes revealed deficits in attention and executive function in one case, with no losses reported in other cognitive domains.

There is limited reported data on MRIgLiTT in the treatment of PVH. One report describes 2 cases where the first patient was seizure free and the second required ATL [6, 15]. Our outcomes for PVH patients are consistent with those previously published, but larger series are needed to draw robust conclusions. In our center, SEEG-RFTC is traditionally considered, as it enables a precise identification of the SOZ, and performing lesions on epileptogenic areas, which are sometimes larger than the identified lesion.

It remains important to note that the technique has a learning curve to achieve its full potential effectiveness while minimizing damage to nearby structures. Postoperative complications after MRIgLITT procedures depend on the location, but as in open surgeries, a frequent problem is a visual field defect [4, 45]. It is usually related to fiber misplacement or heat spread beyond the limits of our target. In our experience, limiting heat exposure along the posterior hippocampus (avoiding optic radiations) by marking the posterior limit of the ablation at the level of the lateral mesencephalic sulcus in the coronal plane, considerably reduces the risk while achieving similar seizure-free outcomes.

Despite conflicting data, tend to be accepted that neuropsychologically results vary depending on the type of surgery, location and extent of resection, as well as dominant hemisphere involvement. Comparing neuropsychological assessment, the incidence of cognitive deficit seems to be significantly lower to the open surgery groups [12]. In our sample, most patients did not experience a significant decline in cognitive performance, consistent with findings from other studies that conclude cognitive deficits after MRIgLITT are modest. Specifically, verbal memory processes were primarily affected, with 26% of our patients showing impairment at the one-year follow-up, aligning with reports in the literature [9, 11, 51].

While its significantly higher cost compared to open procedures remains a major limitation, MRIgLITT has proved to be a safe and effective treatment in patients with lesional drug-resistant epilepsy. It is a well-tolerated procedure that reduces hospitalization time, postoperative pain, and appears to yield better neuropsychological outcomes [14, 41]. It should be considered as an option for patients with well-localized SOZ that are amenable in size and location to ablative treatment. However, depending on the pathology and lesion location the epileptogenic network also plays a role in determining the probability of seizure freedom, in addition to volume. This can be observed in pathologies, such as HH or complex PVHs.

This study is limited by its small sample size and heterogeneity, which precludes significant conclusions and the variability between each of the MRI diagnosis subgroups. Therefore, larger studies, probably prospective multicenter, with longer follow-up periods are needed to draw definitive conclusions. Sharing data is crucial to enhance our understanding of this technique and improve patient selection criteria.

## Supplementary Information

Below is the link to the electronic supplementary material.

Supplementary Material 1 (DOCX 36.5 KB)

## Data Availability

No datasets were generated or analysed during the current study.

## Abbreviations

*ASM*: Anti-seizure medication
*ATL*: Anterior temporal lobectomy
*BRV*: Brivaracetam
*CBZ*: Carbamazepine
*CLB*: Clobazam
*CT*: Computed tomography
*DRE*: Drug-resistant epilepsy
*DZP*: Diazepam
*FCD*: Focal cortical dysplasia
*HS*: Hippocampal sclerosis
*HH*: Hypothalamic hamartoma
*LCM*: Lacosamide
*LEV*: Levetiracetam
*LTG*: Lamotrigine
*MRI*: Magnetic Resonance Imaging
*MRIgLITT*: Magnetic Resonance Imaging (MRI)-guided Laser Interstitial Thermal Therapy
*OXC*: Oxcarbazepine
*PB*: Phenobarbital
*PER*: Perampanel
*PET*: Positron emission tomography
*PGB*: Pregabalin
*PVH*: Periventricular heterotopia
*SCB*: Eslicarbazepine
*SEEG*: Stereotactic electroencephalography
*SEEG-RFTC SEE*: SEEG- radiofrequency thermocoagulation
*SPECT*: Single-photon emission computed tomography
*SOZ*: Seizure onset zone
*TPM*: Topiramate
*TSC*: Tuberous sclerosis complex
*ZNS*: Zonisamide
*VEEG*: Video electroencephalography
*VPA*: Valproic acid
