# Supplementary material for: MRI-guided laser interstitial thermal therapy in epilepsy: indications, technique and outcome in an adult population. A single-center data analysis
Source: Acta Neurochir (Wien). 2025 Feb 8;167(1):39. doi: 10.1007/s00701-025-06429-3 (PMC11807016; doi:10.1007/s00701-025-06429-3)
Supplement: Supplementary file 1 — Supplementary Material 1 (DOCX 36.5 KB) [file 701_2025_6429_MOESM1_ESM.docx]

**SUPPLEMENTARY TABLES**

| **Cognitive domains** | **Attention** | **Executive Function** | **Verbal Memory** | | **Visual Memory** | **Language** | **Visospatial Skills** | |
| --- | --- | --- | --- | --- | --- | --- | --- | --- |
| **Test & variables** | **Digit forward** | TMT-B | FCSRT-A immediate free recall | | ROCF immediate recall | **Boston Naming Test** | JLO | |
|  | Digit backward | **Stroop interference** | FCSRT-A immediate total recall | | **ROCF delayed recall** | Verbal Fluency (animals) | **VOSP position discrimination** | |
|  | TMT-A | TOL-Dx correct problem | FCSRT-A delayed free recall | | ROCF recognition | Verbal Fluency (/p/) | VOSP number location | |
|  | SDMT | TOL-Dx initiation  time | **FCSRT-A delayed total recall** | |  |  |  | |
|  |  | TOL-Dx resolution time |  |  | | | |  |

**Table 4. Neuropsychological assessment protocol (test and variables) according to cognitive domains.** FCSRT: Free and Cued Selective Reminding Test; JLO: Judgment Line Orientation; ROCF: Rey-Osterrieth Complex Figure; SDMT: Symbol Digit Modalities Test; TMT: Trail Making Test; TOL-Dx: Tower of London Drexel Version; VOSP: Visual Object and Space Perception battery. The tests used for the analysis of the RCI in this study are highlighted in bold.

| Cognitive Dm | Attention | | | Executive Function | | | Verbal Memory | | | Figural Memory | | | Language | | | Visuospatial Skills | | |
| --- | --- | --- | --- | --- | --- | --- | --- | --- | --- | --- | --- | --- | --- | --- | --- | --- | --- | --- |
| ID | **T_0_** | **T_2_** | **RCI** | **T_0_** | **T_2_** | **RCI** | **T_0_** | **T_2_** | **RCI** | **T_0_** | **T_2_** | **RCI** | **T_0_** | **T_2_** | **RCI** | **T_0_** | **T_2_** | **RCI** |
| 1 | 7 | 8 | 1.00 | 70 | 58 | -1.66* | 14 | 14 | -0.21 | 21 | 16.5 | -1.14 | 53 | 51 | -0.84 | 20 | 20 | 0.20 |
| 2 | NA | NA | NA | 51 | 49 | -0.53 | 8 | 10 | 1.08 | 13.5 | 9 | -1.14 | NA | NA | NA | NA | NA | NA |
| 3 | 5 | 5 | 0 | 27 | 38 | 0.95 | 8 | 9 | 0.43 | 17 | 12 | -1.26 | 38 | 39 | 0.13 | 19 | 18 | -1.47 |
| 4 | 6 | 5 | -1.00 | 30 | 36 | 0.38 | 12 | 11 | -0.85 | 10 | 9.5 | -0.18 | 53 | 50 | -1.16 | 20 | 20 | 1.20 |
| 5 | 6 | 6 | 0 | 43 | 26 | -2.24* | 15 | 11 | -2.80* | 10.5 | 10 | -0.18 | 46 | 20 | -2.45* | 20 | 17 | -4.81* |
| 6 | 5 | 5 | 0 | 46 | 47 | -0.18 | 15 | 15 | -0.21 | 14 | 19 | 1.14 | 28 | 29 | 0.13 | NA | NA | NA |
| 7 | 4 | 5 | 1.01 | 30 | 39 | 0.73 | 14 | 15 | 0.43 | 11 | 17.5 | 1.51 | 38 | 43 | 1.41 | 20 | 19 | -1.47 |
| 8 | 5 | 3 | -2.01* | 56 | 51 | -0.87 | 16 | 14 | -1.5 | 14 | 18 | 0.9 | 51 | 39 | -4.06* | 20 | 20 | 0.2 |
| 9 | 7 | 6 | 1.01 | 38 | 33 | -0.87 | 12 | 11 | -0.86 | 13 | 18 | 1.14 | 52 | 49 | -1.16 | 20 | 20 | 0.2 |
| 10 | 6 | 4 | 1.01 | NA | NA | NA | NA | NA | NA | 13.5 | 18 | 1.02 | NA | NA | NA | NA | NA | NA |
| 11 | 7 | 7 | 0 | 40 | 23 | -2.24* | 13 | 13 | -0.21 | 17 | 17 | -0.06 | 28 | 31 | 0.77 | 20 | 20 | 0.2 |
| 12 | 8 | 5 | -3.01* | 66 | 64 | -0.53 | 16 | 16 | -0.21 | 22 | 30 | 1.87 | 56 | 56 | -0.2 | 20 | 20 | 0.2 |
| 13 | 6 | 5 | -1.01 | 47 | 48 | -1.61 | 16 | 16 | -0.21 | 19.5 | 25 | 1.26 | 57 | 59 | 0.45 | 19 | 20 | 1.87 |
| 14 | 7 | 6 | -1.01 | 51 | 41 | -1.44 | 16 | 16 | -0.21 | 27 | 22.5 | -1.14 | 50 | 52 | 0.45 | 19 | 20 | 1.87 |
| 15 | 4 | 4 | 0 | 22 | 45 | 2.32 | 15 | 6 | -6.03* | 15.5 | 11 | -0.14 | 54 | 50 | -1.49 | 18 | 20 | 3.54 |
| 16 | 5 | 5 | 0 | 24 | 30 | 0.38 | 13 | 10 | -2.15* | 17.5 | 20 | 0.54 | 43 | 42 | -0.52 | 19 | 20 | 1.87 |
| 17 | 5 | 6 | 1.01 | 51 | 68 | 1.65 | 15 | 12 | -2.15* | 19 | 20 | 0.18 | 53 | 50 | -1.16 | 20 | 20 | 0.2 |
| 18 | 7 | 7 | 0 | 53 | 56 | 0.04 | 16 | 15 | -0.86 | 19.5 | 18 | -0.42 | 49 | 48 | -0.52 | 20 | 20 | 0.2 |
| 19 | 5 | 5 | 0 | 36 | 31 | -0.87 | 15 | 11 | -2.80* | 16.5 | 19 | 0.54 | 31 | 31 | -0.2 | 20 | 19 | -1.47 |
| 20 | 5 | 6 | 1.01 | 57 | 58 | -0.18 | 16 | 15 | -0.86 | 29.5 | 27 | -0.66 | 53 | 52 | -0.52 | 20 | 20 | 0.2 |
| 21 | 7 | 7 | 0 | 58 | 51 | -1.1 | 16 | 14 | -1.50 | 25 | 23.5 | -0.42 | 56 | 51 | -1.81* | 20 | 20 | 0.2 |
| 22 | 4 | 4 | 0 | 28 | 35 | 0.50 | 14 | 9 | -3.44* | 16.5 | 13.5 | -0.78 | 36 | 34 | -0.84 | 19 | 20 | 1.87 |
| 23 | 5 | 5 | 0 | NA | NA | NA | 16 | 16 | -0.21 | 13.5 | 11.5 | -0.54 | 26 | 29 | 0.77 | 19 | 18 | -1.47 |
| 24 | 5 | 4 | -1.01 | 44 | 48 | 0.15 | 15 | 13 | -1.50 | 18.5 | 12.5 | -1.51 | 45 | 38 | -2.45* | 20 | 20 | 0.2 |
| 25 | 5 | 5 | 0 | 3 | 9 | 0.38 | 8 | 2 | -4.09* | 7 | 1 | -1.51 | 35 | 36 | 0.13 | 18 | 20 | 3.54 |
| 26 | 4 | 4 | 0 | 23 | 31 | 0.61 | NA | NA | NA | 17 | 9 | -1.99* | NA | NA | NA | 18 | 19 | 1.87 |
| 27 | 7 | 6 | -1.01 | 50 | 45 | -0.87 | 13 | 14 | 0.43 | 17.5 | 15 | -0.66 | 53 | 51 | -0.84 | 20 | 20 | 0.2 |
| 28 | 6 | 5 | -1.01 | 58 | 49 | -1.33 | 15 | 11 | -2.80* | 14 | 2 | -2.95* | 49 | 48 | -0.52 | 20 | 20 | 0.2 |

**Table 5.** Neuropsychological descriptive data of pre- and post-LiTT raw scores and RCI for each patient, according to cognitive domain (Cognitive Dm). T_0_: Pre-surgical assessment; T_2_: 1-year follow-up; *: Reliable Cognitive loss (RCI<-1.64).
